# Barriers to accessing and receiving antenatal care: Findings from interviews with Australian women experiencing disadvantage

**DOI:** 10.1111/jan.15724

**Published:** 2023-06-27

**Authors:** Sarah V. Penman, Ruth M. Beatson, Elizabeth H. Walker, Sharon Goldfeld, Carly S. Molloy

**Affiliations:** ^1^ Murdoch Children's Research Institute Parkville Victoria Australia; ^2^ University of Melbourne Parkville Victoria Australia; ^3^ North Western Melbourne Public Health Network Parkville Victoria Australia

**Keywords:** barriers, health behaviour, health care, access to, interviews, maternity care, nursing, antenatal care, qualitative methods, social inequity, users' experiences

## Abstract

**Aim:**

To identify the barriers associated with inadequate antenatal attendance by disadvantaged women in Australia and to further explore how these barriers are experienced by this population group.

**Design:**

A qualitative descriptive study utilizing semi‐structured interviews and thematic analysis.

**Methods:**

Interviews were conducted with 11 pregnant women who self‐identified as experiencing disadvantage, purposively sampled from a local government area of Victoria, Australia, characterized by socio‐economic disadvantage. Data were collected from February to July 2019.

**Results:**

Study participants reported a range of barriers to receiving timely and adequate antenatal care (ANC). For several women, a combination of personal (e.g., emotions, knowledge), health service provision (e.g., limited access to continuity of care provider and continuity of information, inflexible scheduling, difficulty travelling, staff attitudes), and broader social‐contextual factors (e.g., financial situation, language, cultural norms) were ultimately insurmountable. Whereas some barriers were experienced as hassles or annoyances, others were unacceptable, overwhelming, or humiliating.

**Conclusion:**

Women experiencing disadvantage in Australia value ANC but face multiple and complex barriers that undermine timely and regular access.

**Implications for the Profession and/Patient Care:**

A wide range of strategies targeting barriers across multiple levels of the social‐ecological environment are required if ANC attendance rates are to improve and ultimately redress existing health disparities. Various continuity of care models are well‐placed to address many of the identified barriers and should be made more accessible to women, and particularly those women experiencing disadvantage.

**Impact:**

Antenatal care appointments promote the health of women and their babies during pregnancy, but for many women, particularly those experiencing disadvantage, access is delayed or inadequate. ANC providers play a critical role in facilitating timely and adequate care. Health service practitioners and management, and health services policymakers need to understand the complexity of the barriers women encounter. These stakeholders can utilize the findings reported herein to develop more effective strategies for overcoming multiple and multi‐level barriers.

**Reporting Method:**

The study is reported in accordance with the relevant EQUATOR guidelines: the standards for reporting qualitative research (SRQR) and consolidated criteria for reporting qualitative research (COREQ).

**Patient or Public Contribution:**

No patient or public contribution.

## 1 INTRODUCTION

Antenatal care (ANC) is a critical platform for identifying and managing conditions that if left untreated can cause maternal and perinatal morbidity and mortality (McCauley et al., 2022). Access to high quality ANC leads to improved birth outcomes (Sandall et al., 2016), and has protective effects for women experiencing various forms of disadvantage that place them at higher risk of adverse birth outcomes (Rayment‐Jones et al., 2021). Thus, ensuring equitable access to high quality ANC is one way of addressing some of the complex social determinants of health experienced by women and infants with low economic resources (Sutherland et al., 2012).

In Australia, ANC is delivered via a universal health platform intended to optimize maternal health and foetal development, and minimize adverse outcomes, for all women and their infants. Timely and regular attendance at ANC consultations with qualified healthcare providers (typically midwives, nurses, and doctors) is associated with: fewer and more effective interventions, and prevention of poor maternal and child outcomes, such as maternal mortality, stillbirth and other perinatal deaths (Australian Institute of Health and Welfare, 2018; Miller et al., 2018). The Australian Pregnancy Care Guidelines recommend that all women attend a first antenatal consultation within the first trimester, and a minimum of 6–7 subsequent consultations in total (Department of Health, 2021). This is consistent with other international best practice guidelines (National Institute for Health and Care Excellence, 2021; World Health Organization, 2016).

Antenatal care consultations provide critical opportunities to identify and manage a variety of risk factors (e.g., maternal smoking, obesity, poor diet, stress and depression) that are associated with poor foetal and later child physical and psychosocial development outcomes (Downe et al., 2009). Many risk factors are more common among women experiencing disadvantage (Australian Institute of Health and Welfare, 2020; Mackenbach, 2015). Recognizing the multifaceted nature of disadvantage, we define it broadly as any financial, cultural, ethnic, legal, educational, social, or health circumstances that reduce the likelihood of an individual experiencing good quality of life (Goldfeld et al., 2018). Examples of such circumstances include poverty, unemployment, homelessness, housing instability, rural or remoteness, lack of education, social isolation, family violence, cultural and linguistic diversity, physical or mental illness/disability. Ensuring that women from disadvantaged backgrounds receive early and regular ANC provides a critical opportunity to redress existing health disparities.

## 2 BACKGROUND

Previous research shows women experiencing disadvantage (e.g. low socioeconomic, rural, ethnic minority status) receive fewer ANC appointments and commence care later in their pregnancy compared with non‐disadvantaged women (Australian Institute of Health and Welfare, 2018). For example, the Australian National Perinatal Data Collection (Australian Institute of Health and Welfare, 2018) indicates that only 70.6% of mothers living in the most disadvantaged areas attend their first ANC appointment in the first trimester, compared with 77.4% of mothers living in the most advantaged areas. Similarly, data from the nationally representative Longitudinal Study of Australian Children indicates that only 66% of pregnant mothers experiencing disadvantage receive the recommended number of consultations prior to their child's birth, compared with almost 72% of mothers experiencing less disadvantage (Molloy et al., 2019). In order to address and minimize existing disparities in health access and outcomes, it is important to understand the barriers that prevent women from disadvantaged groups receiving timely and adequate ANC.

Research investigating disadvantaged women's experiences in accessing and receiving ANC identifies a range of complex barriers that have been categorized in various ways (Bellerose et al., 2022; Downe et al., 2009; McLeish & Redshaw, 2019; Phillippi, 2009). Personal or psychological barriers have included negative emotions, maternal motivations, and knowledge of pregnancy or ANC services. Other barriers relate to issues with the provision of ANC (e.g., clinic or hospital setting and procedures, healthcare providers, and the broader healthcare system), or broader social‐contextual factors (e.g., social issues, cultural norms, language differences, and economic circumstances) (Bellerose et al., 2022; Downe et al., 2009; McLeish & Redshaw, 2019). As recognized in one review, the decision to access ANC follows a process whereby women weigh up and balance out “personal issues and circumstances within their social context, and in the context of the care provision they anticipate and encounter” (Downe et al., 2009, p. 526).

Several international reviews identifying ANC attendance barriers have focused on disadvantaged populations (Downe et al., 2009), such as women experiencing homelessness (McGeough et al., 2020), low socioeconomic status (Bellerose et al., 2022; Origlia et al., 2017), or social risk factors (Rayment‐Jones et al., 2019). Others have investigated the experiences of migrants (Fair et al., 2020; Santiago & Figueiredo, 2015), and asylum seekers (Frank et al., 2021). Though the extant literature provides valuable insights into the breadth and complexity of ANC attendance barriers experienced by women from disadvantaged backgrounds, in high‐income countries most research has been conducted in North America and the United Kingdom (Heys et al., 2021). While Australia is similar to these countries in many respects, the healthcare systems (particularly in North America) are very different, and may plausibly result in women experiencing different barriers to ANC. Therefore, there is a need for qualitative research specifically focusing on ANC attendance barriers experienced by women in Australia. However, there is a lack of data in this area.

A small number of Australian studies have explored the ANC experiences of Aboriginal women (Seear et al., 2020; Simpson et al., 2020), those from refugee or migrant backgrounds (Carolan & Cassar, 2007; Owens et al., 2016; Stapleton et al., 2013), and adolescent mothers in rural or regional areas (Wong Shee et al., 2021). Two studies show indigenous women experience difficulties with the distance of ANC services from their homes, fear and stigma, and a lack of access to culturally safe ANC (Seear et al., 2020; Simpson et al., 2020). Similarly, women in Australia with refugee and migrant backgrounds face difficulties with inconvenient clinic locations, communication problems and poor staff rapport (Carolan & Cassar, 2007; Stapleton et al., 2013). Teenage mothers in rural and regional areas also report geographical, financial, social, and scheduling barriers to receiving ANC (Wong Shee et al., 2021).

## 3 THE STUDY

### 3.1 Aim(s) and objective

Extending our understanding of the barriers specific to Australian women experiencing disadvantage is necessary to better inform the efforts of Australian policy makers and ANC providers seeking to overcome ANC attendance barriers, increase equitable access to ANC, and ultimately reduce inequitable health outcomes. Therefore, the present study aims to address this gap in evidence to inform policy by identifying the barriers associated with inadequate antenatal attendance among disadvantaged women in Australia and exploring how these barriers are experienced by this population group.

## 4 METHODS/METHODOLOGY

### 4.1 Design

This study utilized qualitative description methodology and semi‐structured interviews with a purposive sample of 11 women residing in areas characterized by low socioeconomic status. Qualitative description is used to describe and understand a phenomenon or perspectives of which little is previously known (Bradshaw et al., 2017). In addition, it is often used to provide clear information on how to improve clinical practice (Bradshaw et al., 2017). Data analysis involved development of a framework for categorizing barriers into broad domains identified in previous literature (Downe et al., 2009; McLeish & Redshaw, 2019; Phillippi, 2009) and thematic analysis of participant responses to facilitate a rich and in‐depth understanding of the contextual complexity within which women experience barriers to accessing and receiving ANC.

### 4.2 Sampling and recruitment

A purposive sampling method was used with recruitment efforts primarily targeting a local government area (LGA) characterized by low Socio‐economic Indexes for Areas (SEIFA) (Brimbank Shire, Melbourne, Australia), an indicator of relative disadvantage. More than 30 community groups and organizations within this area were approached and encouraged to advertise the study. These included maternal and child health centres, libraries, playgroups, community house organizations, kindergartens and preschools, general practice and allied health clinics. A variety of promotional methods were deployed (e.g., distributing flyers, announcements at meetings, postings on websites or intranet, email and word of mouth). Snowballing was also used with service providers and participants encouraged to promote the study to other potentially interested organizations or families, respectively, by sharing researcher contact details or the link to an eligibility screening questionnaire. Women were not recruited from birthing wards, as previous research shows realistic reflection on ANC and processing of experiences takes time (Roberts et al., 2017).

Recruitment was open from February to July 2019, with women interested in the study given the opportunity to complete an online eligibility screening survey. Of 13 women who met eligibility criteria, described below, 11 scheduled an interview, with all completing it. Two eligible women declined interviews but did not give reasons.

### 4.3 Population and sample

Participant age ranged from 23 to 40 years. All participants reported experiencing some form of disadvantage during pregnancy and attended publicly funded ANC though a hospital, GP, or midwife caseload programme. Ten were from LGAs in metropolitan Melbourne where SEIFA indicates relative disadvantage, and one was from regional Victoria. Seven were single mothers. Four had a non‐English speaking background, including the participant from regional Victoria. Three participants required a translator.

### 4.4 Inclusion and exclusion criteria

Eligible participants were women who had given birth and experienced disadvantage within the previous 2 years. There were no restrictions with respect to parity, risk factors, or previous birth experiences. Eligibility was not restricted to women who had attended less than the recommended number of ANC consultations; we reasoned that woman who had received an adequate number of ANC appointments may nevertheless have experienced barriers to attendance and could provide useful information about these experiences. Eligibility criteria were assessed via screening questionnaire or interview depending on participant preference. Disadvantage could be self‐identified and was broadly defined to include any adversity arising from financial, cultural, ethnic, legal, health or social circumstances.

Women were not eligible to participate if the majority of their pregnancy had occurred outside Australia or if a translator was not available at the time of interview (through community or ANC services) to assist women not fluent in spoken English.

### 4.5 Data sources/collection

Semi‐structured interviews were guided by a series of open‐ended questions intended to elicit participant beliefs about, and experiences of, ANC (see Table 1). The interview guide was developed to include explicit probes about (1) any personally experienced difficulties in attending ANC appointments, and (2) barriers participants perceived other families might face. Although spontaneous generation of additional questions was also allowed in pursuit of interesting material that emerged, the guide was not changed during the course of data collection. Ten interviews were conducted in person (either in community settings such as libraries or in participants homes) and one by telephone. Three interviews were mediated by translators. In several cases, other family members (e.g., children, partners) or friends were present. Interview duration ranged from 21 to 107 min (Mean 52, Med = 58). Interviews were digitally recorded via mobile phone and transcribed verbatim by an external transcription service (Rev.com).

**TABLE 1:** Semi‐structured interview guide.

Introduction
• Build rapport, briefly discuss family structure
• Explain the purpose, scope, procedure regarding recording device, rights and limits to confidentiality, answer preliminary questions, and confirm informed consent

Interview questions
1. Could we start with what you know about Antenatal Care?
• What happens in appointments?
• What is the purpose of appointments?
2. What is your opinion of the Antenatal Care Service?
• Do you think it is important?
• Do you think attending is worthwhile?
3. What is your experience with Antenatal Care Service?
• Did you know about the service?
• Did you make any appointments?
• Did you attend any appointments?
4. Did you manage to make all of your appointments?
• Why not?
• What got in the way?
5. You mentioned that ______ was an issue for you, can you tell me more about that?
(*Explore barriers*)
6. Do you think there is anything that could be done to help yourself and other families make it to more appointments?
• To overcome _______ you mentioned?
• What would make it easier?
(*Overcoming barriers*)
7. We know it can be difficult for some families in the community to make it to any or all of their appointments … do you have any thoughts on why this might be?
(*Perception of barriers faced by others*)
8. That is the end of my questions. Is there an aspect of your care we have not touched on, or anything you would like to add?
(*Wrapping up and giving an open floor to participant*)

### 4.6 Data analysis

The first author (S.V.P.) reviewed all transcripts for accuracy, uploaded the files to NVivo (QSR International Pty Ltd, 2015), and conducted a codebook thematic analysis with an interpretive approach (Braun et al., 2019). The unit of analysis was individual comments (i.e., phrases, sentences) indicating an ANC attendance barrier or barriers. The early stages of analysis stayed close to women's meanings and descriptions of their experiences, and the first phase of coding identified any barrier to access or attendance at appointments according to women's accounts. The researcher then worked through the transcripts multiple times to examine each example of a barrier coded in the initial phase, considered to what extent it constituted a barrier, and identified the relevant category. Barriers identified in previous literature (Downe et al., 2009; McLeish & Redshaw, 2019; Phillippi, 2009) were used as the basis for 11 categories that emerged during the interviews: (1) emotions, (2) motivations, (3) knowledge of pregnancy or ANC, (4) the health clinic or hospital, (5) healthcare staff, (6) healthcare system, (7) cultural norms or standards, (8) social issues (9) economic factors, (10) immigration status, and (11) language issues. Single sentences could contain multiple barriers and each barrier was coded according to one of the above categories. Where additional barriers were identified, there was provision to add these to the coding framework. While the deductive codebook approach to analysis addressed the first part of the research question (i.e., which barriers Australian women experience), elaborative description of barriers addressed the second part (i.e, developing a deeper understanding how women experience barriers).

### 4.7 Ethical considerations

The study was approved by Royal Children's Hospital Human Ethics Committee and performed in accordance with the Declaration of Helsinki (World Medical Association, 2013). In compliance with ethics approval raw data are not publicly available. It will be stored electronically and then destroyed either 7 years following project completion or 5 years from publication of results.

All participants provided informed written or verbal consent, and none withdrew from the study at any stage. Interviews were conducted at participant homes or an appropriate community location. Interview participants were given a $20 supermarket voucher as a token of appreciation for their time. Funding organizations had no role in the collection, analysis or interpretation of data, nor in the decision to publish the findings.

### 4.8 Rigour

To ensure analytic rigour, a second coder (E.H.W.) applied the deductive codebook analysis to a randomly selected subset of four interviews. Discrepancies in coding were discussed until consensus was reached, and the final coding categorisations were checked by another author (R.M.B.). Discussion of double‐coding showed strong consensus with regard to code categorisations and overall interpretation of contextual complexity. Owing to research resourcing, it was not possible to seek participant feedback (i.e., comment or corrections) on transcripts or findings.

The study was designed by a female Caucasian Australian researcher (C.S.M.) with postdoctoral experience in psychology and paediatrics. All interviews were conducted by the first author (S.V.P.), a female Doctor of Medicine student enrolled at the University of Melbourne and completing the research component of her course at the Murdoch Children's Research Institute. The wider research team had established prior relationships with key stakeholders (e.g., ANC and early childhood services in the recruitment area), but the interviewer and analyst (S.V.P.) had no previous relationship with participants. She attempted an unbiased, open minded approach to data collection and analysis, which emphasized participants' perspectives and experiences but also sometimes reflected on her knowledge of the ANC system in Australia.

## 5 FINDINGS

As shown in Table 2, all 11 barrier categories were observed in the interviews and no additional barriers emerged. Critically, barriers did not occur in isolation. Rather, women reported experiencing multiple and overlapping barriers, each of which will be discussed in more detail below.

**TABLE 2:** ANC attendance barriers.

Psychological	Health services and system	Broader social contextual environment
Emotions	Setting	Cultural norms
• Anxiety/fear • Depression • Frustration	• Wait times • Inflexible appointment scheduling • Distance from women's homes	• Stigma associated with youth, prior terminations • Alternative medicine and natural therapies • Concern with cultural safety
Motivations	Staff	Social issues
• Fluctuation and sensitivity	• Lack of rapport or respect for women • Poor communication and collaboration • Conflicting advice • Perceived incompetence	• Lack of social support • Competing family responsibilities • Work‐life imbalance • Family violence
Knowledge	System	Economic factors
• Unsure of attendance benefits • Lack of information about models of care • Lack information about patient rights	• Lack of continuity of care • Lack of interagency collaboration • Inconsistent standards of care	• Low income and financial stress • Additional medical costs • Hidden costs of attendance (transport, parking)
		Immigration status
		• Hindered access to Medicare
		Language
		• Lack of translators
		• Reliance on family for translation

Abbreviation: ANC, antenatal care.

The most frequently discussed barriers related to social and economic factors, clinical and administrative ANC staff, health clinic/hospital settings, and emotions. Barriers relating to immigration status and cultural norms emerged less frequently. Utilizing an overarching theme taxonomy similar to previous research (Mazul et al., 2017), we first describe psychological barriers (i.e., emotions, motivation, knowledge), and then progress to healthcare service and system barriers (e.g., practitioners, services, and systems) and broader social‐contextual factors (e.g., cultural norms, social issues, economic circumstances).

### 5.1 Psychological barriers

#### 5.1.1 Emotions

Several women identified emotional barriers to accessing or receiving ANC. These included difficulties with anxiety, depression, stress and feelings of being overwhelmed. Pregnancy was described as an exhausting, emotionally unstable time, upon which ANC appointments was an additional strain. Some participants suggested avoidance was used as a method of dealing with stress, reflecting that they may have been ‘better not knowing’ about their own medical conditions or those of their unborn baby:

You don't want to hear things that you don't want to hear … Ignorance is bliss, maybe? And especially in pregnancy … it could be pretty scary, I guess, to find out something is not right with your baby. [Participant 6]

For those with pre‐existing mental health conditions, the additional stresses of pregnancy and interacting with the healthcare system could become overwhelming:

I get prenatal depression at the best of times … It's too much, so you're sitting there at home, knowing you had the appointment, and being like, yeah, no, screw ya. I'm not doing it. [Participant 2]

Some emotional barriers were clearly situated within the context of a woman's family relationships, such as with the baby's father. While reflecting on a missed appointment, one participant explained the inner dialogue that had contributed to her not attending:

Negative self‐talk said you're not worth it. It's not worth it. The baby's not loved anyway. His father doesn't want him. His father doesn't want you. You might as well just sit here and rot. [Participant 2]

Anxiety and fear of judgement from practitioners also emerged as emotional barriers. Some participants believed they were expected to know more than they did. This was associated with feelings of otherness and fear of challenging standard practices:

[I felt] really powerless … Because it's just another grade of isolating, I guess. Saying that I don't fit in, I'm not a part of this, and just scared of voicing what I want to say because they might think I'm … I don't know. [Participant 8]

#### 5.1.2 Motivation

Overall, most participants placed significant value on ANC and were highly motivated to attend their appointments, at times overcoming substantial barriers to do so. However, there was a tendency for motivation levels to fluctuate over the course of a woman's pregnancy. This fluctuation corresponded with the health and wellbeing of the mother, the challenges faced in her personal life and the experience of engaging with the ANC system, services, and staff. Six participants described instances where they lacked or questioned their motivation to attend appointments. For some, this resulted in multiple missed appointments, or disengagement from a service altogether.

Motivation was related to a variety of other barriers which often coincided, likely having a cumulative negative effect on attendance. These included unpleasant healthcare experiences, health issues like depression, or lacking information about the benefits of ANC.

As one participant put it:

It takes a lot of different aspects to make the choice to not go to your antenatal appointments, because they are important, and you want to make the best choice for your baby … As someone who's growing a person your whole instinct is to do the right thing for them … I can't imagine [just a single] scenario where I would turn away from medical professionals … It would have to be a multitude of things that made me go, “Actually, you know what I'm not going to do this.” Not today Satan, not today. [Participant 9]

#### 5.1.3 Knowledge of pregnancy and ANC services

Seven women described barriers that could be understood as stemming from a lack of information. In all cases women were aware of their pregnancy early and confirmed this with a GP, usually before 10 weeks, when they were referred to an ANC program. The most prevalent knowledge‐based barrier was a lack of awareness or trust in the benefits of timely and regular ANC. Appointments could feel uninformative and unnecessary:

If I felt like me going to the appointments would've been useful, I would've gone. If I felt like it would've been worth it, worth the effort, I would've gone. But they just felt really arbitrary. [Participant 9]

Other knowledge gaps related to the various models of care available, the workings of the healthcare system, and patient rights within it. These knowledge gaps were associated with apprehensions about the potential consequences of voicing one's concerns or needs, and ultimately lead to discontinuation of attendance for some women:

I just didn't want to have my care changed halfway through so I didn't want to complain in case that option [model of care] was taken away from me. [Participant 9]

### 5.2 Health service and system barriers

#### 5.2.1 Health clinic or hospital

Most participants discussed barriers relating to the ANC setting, including both health clinics and hospitals. Issues associated with ANC settings mainly concerned scheduling of appointments and lack of transportation support. Wait times were a source of considerable frustration for the majority of women. One woman described a situation where she was referred to a pregnancy care unit for emergency investigation, but left without seeing a healthcare professional after waiting 4 h. This resulted in an anxious 3‐week wait for her next routine check‐up, where the woman made no mention of what had happened (for fear of being sent to the unit again) and the practitioner neglected to enquire about it. Long appointment windows (e.g., 8 a.m.–12 p.m.) were or an inability to keep appointment times was frustrating. This was compounded by the discomfort women felt in their changing bodies when they sat for long periods on uncomfortable waiting room chairs. The experience was exhausting for some: “Waiting time is horrible, absolutely horrible … Your back hurts, your legs hurt, the couches are always uncomfortable.” [Participant 1].

Lack of flexibility over appointment days and times was also problematic. Appointments did not consider women's other responsibilities (e.g., transporting children to school), and sometimes resulted in delayed diagnosis or treatment if the clinically designated follow‐up day was already booked out. Rigid appointment streams also meant that women requiring many appointments from different treatment teams had appointments spread across multiple days:

Maybe if some of those appointments had been on the same day, so I didn't have to be traipsing in and out and waiting two hours for a 15‐minute appointment to then be told, ‘Why are you here?’ … Maybe. It was a series of things that happened that led to me [not going] … They never called me. I just stopped going. [Participant 9]

For several participants who did not have access to a vehicle, the distance from home to hospital, and availability or reliability of public transport were considered barriers. For the one rural participant, a lack of transport had serious consequences (i.e., inability to access specialist care following a foetal abnormality diagnosis). When public transport was available, it was often not pleasant, and sometimes completely unacceptable:

I got really bad travel sickness … I got kicked off the bus once while I was pregnant … there's nowhere for me to sit down … I warned the bus driver that I was not feeling good, because I'd just been to an appointment, so I'd been poked and prodded … half‐way to Diggers Rest station, I'm puking all over the place. It's horrible … I felt so bad, I was crying … I'm like seven or eight months pregnant … I didn't have a choice, and I do not travel well when sick … we get to Diggers Rest, he opens the door, is like “Get off my bus,” and he was angry … I'm in tears because we couldn't get home. [Participant 1]

Difficulties with transport also interacted with health issues. For some women, travel to appointments could result in considerable and sometimes overwhelming pain or discomfort. As no transportation support was supplied by the hospital, incapacitated women (for example, one who had a broken leg) depended on social support, which was not always reliable or available.

#### 5.2.2 Healthcare staff

Most participants discussed barriers relating to healthcare staff. Lack of communication between practitioners, conflicting advice and perceived incompetence were detrimental to attendance; these experiences eroded both trust in the practitioner and value attached to appointments: “It was a different one [midwife] every time,” so if there's something you've had to make a note of before, then you get requestioned every time, and you're like, “Don't you just read your bloody notes?” [Participant 8].

Some women felt the need to ‘stand up’ for themselves and the care they wanted. They felt staff did not to genuinely consider their wishes. Several women found they lacked the confidence to question the care they received with their first pregnancy, but became more assertive with subsequent ones. Women wanted to explore options and be listened to:

[I would have liked more] listening. Exploring more options … [To get the care I wanted] I just kept putting my foot down, foot down, foot down, foot down, foot down … For some women that might be actually a reason that you don't want to go because you're like “I don't want to have to face that, fight that, say that”. [Participant 6]

Some women felt they were not treated as an individual. They wanted woman‐centred care tailored to their individual circumstances, rather than broad diagnostic criteria and practice policy: “It was as if I was just an item in the production line” [Participant 10]

I asked the questions and I just get them reading from the manual … I'm like, “Yes but how is this relevant to me?” … I think it felt like there was a spectrum, and at no stage in any of those appointments was I treated like I was on a spectrum; you have it or you don't. I said, absolutely I've got it. I'm not questioning my diagnosis. I'm questioning the approach to treatment. [Participant 9]

Many women experienced a lack of emotional support from ANC staff. They described feeling a lack of human connection, respect, compassion or encouragement to discuss their emotions. Some felt rushed by practitioners whom they perceived had inadequate time or energy to expend on their care:

It was like they hated being there and they hated you for being there. And it was very rushed, nothing was explained, and I guess again they wanted to tick boxes and get you out of there as fast as possible. [Participant 10].

Or as another woman put it:

It's like with some people you're a bit of a number. You can hear them work out when they're inducing people to suit their schedule, rather than let the baby come naturally or whatever. They just want to get things done according to them … [You want to feel] that you are important, that you are intelligent, and you have feelings … I think they can just get so busy in their jobs they forget to be human with people … [Participant 8]

Participants also described feeling uncomfortable about asking questions and thinking they were expected to know more than they did. For some, this led to appointments feeling uninformative:

It seemed like a waste of time … I probably would have stopped going because it wasn't pleasant … You didn't get any answers, they just did testing and that's it … You couldn't ask questions … I feel like I would have [known] as much as I did coming into the appointments as I did leaving. [Participant 10]

Some women felt unfairly judged and disrespected: “[The doctors] are very not understanding, and because they're in [suburb name], they look at everybody as if they're drug users.” [Participant 2].

Others discussed how previous negative experiences could have detrimental effects on attending ANC consultations for subsequent pregnancies. One woman described a particularly distressing experience that had occurred during a much earlier pregnancy (17 years ago) where a male doctor's behaviour was inappropriate, disrespectful, and potentially an abuse of power. She said the doctor conducted a breast examination that was unnecessary, and in the presence of another male (student) doctor. As the woman explained, when she would have consented to the student doctor attending the appointment, she had not known she would be instructed to remove her top: “I felt violated and like they just did it because they had the power to do that. There was no reason.” [Participant 6].

#### 5.2.3 Health care systems

Several women discussed barriers associated with the healthcare system. They identified issues with regulations and guidelines for practice, diagnostic standards, and negative prior experiences with the system. The rotating system of practitioners in public hospitals was considered a barrier for some women; several noted varying experiences from 1 week to the next, with the exception of those in the midwife caseload program. Women in the midwifery caseload program reported positive experiences with their practitioners, valuing the relational continuity and advocacy provided by the midwife. As one woman put it: “it was really good to have that backup, somebody that I knew, somebody who was on my side, and was able to be like, yeah no, there is no reason to induce you right now” [Participant 11].

Rotating staff rosters generally left women feeling frustrated with a lack of continuity of care; they were unable to develop relationships, needed to repeat themselves, and found missed appointments were often not followed up. Several women noted that lack of practitioner continuity meant providers were often not aware of the complexity of a woman's situation which could include sequential or concurrent difficulties of significant magnitude. In four cases, women described having very complex personal situations, for example simultaneously dealing with a mental health problem, abandonment or domestic violence from intimate partners, a lack of transport, housing insecurity, financial pressures and responsibility for multiple children or adolescents, and a clinically high‐risk pregnancy or additional health concerns accompanied by extensive physical pain. Another system‐level barrier related to a lack of effective inter‐agency collaboration and consistency. Several women voiced frustrations with perceived poor interdisciplinary communication, inconsistent care, and diagnostic standards both between and within hospitals:

I just couldn't see why I was doing them [ANC consultations] especially when I was getting such conflicting information every second week. One Thursday I'd see the GD [gestational diabetes] team, and the next Thursday I'd see the doctor, and they'd tell me different things. About big stuff. [Participant 9]

Having different specialty appointments scheduled on different days was frustrating for some women. Some challenged their care if they were aware of other women attending different hospitals with similar clinical risk factors receiving different or less intensive treatment plans. Some women with additional difficulties (e.g., mental health) were not provided referrals that would help remove some barriers to ANC. As one woman put it:

There are a lot of great services out there, but you've really got to fight to get them. And you've got to have a lot of either both time and mental energy to do the research … if your anxiety is like mine, where one thing that I really struggle with and can turn into a panic attack is having to call somebody to make an appointment. [Participant 11]

Some women felt frustrated at the number of appointments required. This was particularly the case towards the end of pregnancy, and among those with health complications or additional clinical risks (e.g., gestational diabetes, hyperemesis gravidarum, obesity, depression). Yet others wanted appointments earlier in pregnancy, but were often not booked into their first appointment until they were approaching 20 weeks: “So I reckon why wait until you're so many months to start going to these appointments, why not go when you first find out and get a run down?” [Participant 8].

Negative experiences with the healthcare system sometimes led to distrust and avoidance. As one woman put it: “… there's absolutely no chance I will ever step foot in a hospital ever. I would rather have my baby delivered in the street than step foot in a hospital” [Participant 10].

### 5.3 Broader social context

#### 5.3.1 Cultural norms or standards

Mothers described barriers relating to cultural norms or standards and associated stigma. Pregnancy at a young age, medical histories involving terminations, beliefs about alternative practices and cultural safety were discussed:

Every time I travelled and I saw pretty much anyone look at me, I felt judged. I felt like I'm too young to be doing this … not knowing that there are girls from 18 to 25 that are doing the exact same thing I am, made me feel very alone … It was really hard for me. I think if there were younger people who were going [to the group sessions], I probably would have [attended]. [Participant 1]

Another mother described how disclosure of terminations was uncomfortable: “Whatever surgery you've had, I have to write it down, you're never off the hook ever. Not that you need to be off that hook, but it's like you're branded and that's it.” [Participant 8].

Others discussed alternative medicine or natural therapies sub‐culture and how this introduced some scepticism about ANC:

A lot of my friends are very sceptical of medical professionals … [they have] the idea that a woman's body is meant to know what it's supposed to do. I know my baby, I know my body. I don't need to be told … I'd say hippie dippy in the West community. [Participant 9]

Some women also mentioned the importance of cultural safety:

I guess other families are also from migrant backgrounds or have different religions, and just beliefs, and maybe they want someone from their own country to help them, or they want to practice their own traditional birthing ways. [Participant 10]

#### 5.3.2 Social issues

All women discussed social issues as barriers to accessing or receiving ANC. These related to a lack of social support from family or friends, family responsibilities and work‐life balance, and domestic violence. With regard to family responsibilities and domestic work, women noted that caring for other children sometimes hindered attendance at appointments (e.g. when waiting for others to collect children). When mothers took children to ANC appointments, this was not always easy for the parent (e.g., supervising boisterous children in waiting rooms) or well received by practitioners. Mothers also struggled with appointments that clashed with their children's schedules:

… how am I getting my kids to school and then getting to the hospital, in Sunshine from Sunbury by 8 o'clock in the morning, as a single parent? [Participant 2]

Many women also juggled paid work commitments. Though some noted that their workplaces were supportive, work represented an additional factor to negotiate:

It was a lot of appointments … it can be hard to see in the moment the value of that compared with, oh, if I arrive late at work today, then I have to stay late, and that's going to affect … so many things. [Participant 11]

The time‐consuming nature of having multiple responsibilities was weighed up against the value of attending ANC appointments. As one mother stated:

[I'm] a working mum with a dog who needs to be walked, and a child, and a company, and sickness in pregnancy. To make these appointments [it] has got to be worth your while because your time is precious. [Participant 9]

In addition to a lack of social support and having multiple responsibilities, several participants also discussed domestic violence. These women noted that domestic violence could impede attendance at ANC appointments in a variety of ways that intersected (e.g., inability to access finances, negative impact on self‐worth, partner's routine). One woman noted how a partner's control of finances impacted her self‐worth and led to fear of judgement at ANC visits:

I didn't get maternity clothes and that like everybody else… I'd sit there in these tracksuit pants and just look like a [low SES suburb] mum which I am not. I don't take drugs and I don't have tats all over me and slag off down the street, and if someone would glance at me, I would think that that's what they would think of me…There was six weeks where I didn't have a bra that fitted…I had to save up for it all… it was just not important to my partner… obviously he had what he needed and that was it. So you can't spend money. [Participant 8]

Later in the interview, this participant also noted difficulty talking about domestic violence in ANC appointments:

How to talk about domestic violence… [if] you could just hop into this space and no one could touch you, and you could feel safe and protected and know you didn't have to go home and get your stuff.

#### 5.3.3 Economic factors

All women identified the financial circumstances of their lives as barriers. Financial stress was experienced by all, and low income was particularly recognized as a barrier for single mothers and families experiencing unemployment. Financial barriers involved additional medical costs (particularly for those without access to Medicare), maternity clothing, public transport and parking fees or fines (often associated long wait times for appointments). Financial issues were sometimes tied to feeling shame:

You already feel like shit for being low on money that week and when you've got to ring up and go, “Oh I haven't got the money to come.” … You don't tell them the real reason because you feel really small. [Participant 8]

#### 5.3.4 Immigration status

For some women from migrant backgrounds, visa status presented a barrier to accessing Medicare. One participant reported that she could not access Medicare for the first 20 weeks of pregnancy. This resulted in considerable financial costs (consulting the GP, paying for ultrasounds and blood tests etc.), and difficulty accessing hospital‐based ANC appointments: “From the first month's pregnancy [we] don't have any Medicare card. [We] really struggle.” (translated, Participant 4). Despite these difficulties, women who were not fluent in English tended to identify fewer barriers and were more satisfied with their care.

#### 5.3.5 Language

Although four women from non‐English speaking backgrounds were interviewed, none reported having personally experienced language barriers. All had access to some form of translators and written information when required. However, as one mother observed, not all women have access to an appropriate translator:

There was one other woman who was Vietnamese, and they had her husband translate everything for her, which I think raises red flags. If there was anything going on at home that she wanted to talk to her husband about. [Participant 11]

This woman also noted that language barriers could interact with emotions and exacerbate difficulties with giving and receiving information at ANC consultations:

I imagine for women who don't speak English as a first language and struggle with English, that it would also be a pretty depressing thing going and trying to make yourself understood and trying to understand all of these rapid‐fire questions that are coming at you, because there's so many questions constantly. [Participant 11]

## 6 DISCUSSION

This study addresses a critical gap in knowledge about the ways in which disadvantaged Australian women experience barriers to accessing and receiving ANC. Although previous Australian research explored the ANC experiences of indigenous and migrant women (Owens et al., 2016; Seear et al., 2020; Simpson et al., 2020), there is a dearth of qualitative data that specifically reports on barriers encountered by women experiencing other forms of disadvantage (e.g., low socioeconomic status, mental health issues, family violence). Our research shows that women experiencing disadvantage in Australia value ANC, but face multiple and complex barriers that undermine timely and regular ANC. For many women, a combination of personal (e.g., emotions, knowledge), health service provision (e.g., continuity of care, scheduling), and broader social‐contextual factors (e.g., financial situation, language, cultural norms) likely culminated in a raft of sometimes insurmountable barriers.

Overall, these findings are consistent with previous research showing that women from disadvantaged backgrounds experience a range of barriers to accessing ANC. Prior research from North America, UK, Europe and Australia has consistently reported barriers relating to healthcare services and staff (Bellerose et al., 2022; Downe et al., 2009; Heys et al., 2021; Seear et al., 2020). For example, in multiple studies disadvantaged women have frequently expressed feeling a lack of connection to practitioners in a rushed, depersonalized, inflexible setting (Heys et al., 2021; McLeish & Redshaw, 2019). Both prior and present research highlight the need for positive, respectful, and individualized continuity of care.

Other findings contrast with previous research. For example, most participants in the present study were highly motivated to attend appointments, although this fluctuated in the context of other barriers. Previous research identified motivation as a substantive barrier which was at times lacking from the beginning of pregnancy for women experiencing circumstances requiring that they prioritize basic survival (Downe et al., 2009; Origlia et al., 2017; Rayment‐Jones et al., 2019). In contrast to Australian studies with a focus on migrant or refugee populations (Carolan & Cassar, 2007; Owens et al., 2016; Stapleton et al., 2013) the current study found there was less emphasis on language barriers and cultural safety. This is likely to be attributable to differences in sample characteristics. Additionally, the types of cultural norms, beliefs, and standards that have been identified in studies with indigenous Australian women (Seear et al., 2020; Simpson et al., 2020) were different from the types of cultural norms and standards that emerged in the current study. Further research with larger and more diverse samples is needed to more thoroughly investigate variation in the ANC attendance barriers experienced by women from different backgrounds. Similarly, there is a need for research specifically focussing on what Australian women want from ANC, as previous reviews of the literature identify a dearth of research from Australasia (Downe et al., 2016).

### 6.1 Implications for policy and practice

The barriers identified in this study indicate that improving ANC access for women experiencing disadvantage requires multi system‐level strategies that recognize the complexity of the environment within which women seek healthcare. These findings indicate that the current system of traditional ANC clinics may not adequately support many disadvantaged women and their families (Sutherland et al., 2012). Our research suggests strategies to improve ANC attendance will need to address a variety of psychological, health service provision, and broader social‐contextual barriers.

With regard to psychological barriers, the prior and current findings demonstrate a need to better promote both the benefits of timely and regular ANC and the wide range of ANC models available to women in Australia (Owens et al., 2016; Simpson et al., 2020). The results also substantiate the need to better identify and support pregnant women with mental health difficulties, particularly anxiety and depression (Rayment‐Jones et al., 2019; Savory et al., 2022).

Strategies to ameliorate barriers associated with ANC practitioners, the service setting, and health system should ensure that women: feel respected and listened to during consultations, have continuity of healthcare practitioners, are able to schedule consultations with greater flexibility and shorter wait times, can access their preferred model of ANC close to home, and receive appropriate transportation assistance or home visits when needed.

Finally, strategies to address barriers associated with broader social‐contextual issues should: remove financial disincentives to attending ANC consultations (such as parking fees at service settings), reduce stigma associated with variations from prescriptive mainstream cultural norms, recognize the multiple responsibilities women negotiate and facilitate improved access to formal and informal systems of support, and ensure that women from culturally and linguistically diverse communities, regardless of immigration status, are able to access Australia's publicly funded universal health insurance scheme (Medicare), translators, and culturally safe ANC care.

Caseload midwifery, group ANC and community‐based models of care designed to provide continuity of carer and maximize social support are able to address many of the identified barriers and may increase ANC attendance. Certainly, there is evidence that models such as these are not only safe (Catling et al., 2015; Sandall et al., 2016), but improve several birth outcomes for disadvantaged women and their babies (Rayment‐Jones et al., 2021), are associated with higher levels of ANC satisfaction (Forster et al., 2016; Sandall et al., 2016) and with more empowering and respectful care (Allen et al., 2017). For example, Forster et al. (2016) found that compared with women receiving standard care, women randomly allocated to caseload midwifery were significantly more likely to report that, during pregnancy, they were asked if they had any questions, were kept informed, were given a say in decisions, that their concerns were taken seriously, and that midwives were less rushed. Interestingly, there was no difference in the number of antenatal appointments attended in that trial, but this may be attributable to the restricted sample (low risk pregnancies and not especially disadvantaged women). Unfortunately, there is evidence that high demand for models such as caseload midwifery is coupled with limited access (Watkins et al., 2022) that disproportionately affects women experiencing disadvantage (Sutherland et al., 2012).

Though some barriers may best be addressed at the level of government policy (e.g., access to Medicare, location of services), it is worth noting that for most barriers, there are likely to be a range of strategies that individual practitioners and ANC services (departments or organizations) could implement directly. For example, practitioners might consider ways they can personally promote and reinforce ANC attendance (e.g., via dissemination of information about models of care and benefits of attendance, improved rapport, regularly checking whether women are encountering barriers to attendance and connecting them with appropriate support services to address these).

Practitioners must be supported at an organizational level, with services considering how existing policies and procedures (e.g., respectful care policies, cultural competency training) could be improved to build practitioner capacity. Services that give women access to continuity of care provider and allow for longer consultations should increase capacity to sensitively assess and respond empathetically to each woman's individual needs. This can further be enhanced by additional training and supervision to improve workplace culture, and provision of culturally appropriate care.

Service delivery should be tailored to better recognize the complexity of women's circumstances, and facilitate stronger interagency communication and collaborations to address their needs. According to one maternity care classification system, there are at least 11 major models of care offered in Australia, with new models emerging in response to consumer demand and evidence of benefits (Donnolley et al., 2016). Ensuring that health services inform women about the range of care options available and support them to access their preferred models of care would likely go a long way to improving ANC attendance. There is evidence that when models of care are appropriately tailored, uptake is high. This has recently been demonstrated in an evaluation of a culturally responsive caseload midwifery program offered to Australian First Nations women (McLachlan et al., 2022). Previous research with Australian migrant women has similarly demonstrated support for culturally appropriate continuity of care models (Owens et al., 2016).

### 6.2 Strengths and limitations of the work

This study involved a relatively small sample size. As such, it is likely some barriers were missed. Difficulties recruiting participants from disadvantaged backgrounds are well recognized, nevertheless, previous qualitative research shows valuable insights can be obtained even with small samples (Owens et al., 2016). It is worth noting that recruitment efforts were extensive (involving more than 30 organizations, snowballing procedure) and accommodating (conducting interviews at places convenient to participants, financial acknowledgement of time). Moreover, a reasonably diverse sample was recruited with various forms of disadvantage represented (including economic disadvantage, mental health problems, cultural and linguistic diversity, sole parenting). Another strength is that the sample included women who had missed ANC consultations and women who had attended all their consultations despite substantive barriers. Thus, despite the small sample size, a varied range of experiences was nevertheless canvassed.

As almost all participants accessed ANC in socio‐economically disadvantaged areas of metropolitan Melbourne, the findings are most applicable to similar metropolitan settings within the Australian healthcare system. Though the barriers identified by the one regionally located participant were consistent with those of her metropolitan counterparts, further research is needed to explore ANC attendance barriers faced by women living outside capital cities and experiencing disadvantage.

### 6.3 Recommendations for further research

In addition to demonstrating the range and complexity of barriers that hinder attendance at ANC consultations for women experiencing disadvantage, this study suggests several important directions for future research. Following identification of ANC attendance barriers, the next steps for research will be to identify, develop, implement, and evaluate potential strategies to increase ANC consultation attendance. Previous reviews of interventions to increase ANC utilization identify a variety of potential strategies such as media campaigns, home visits, and financial incentives (Mbuagbaw et al., 2015). However, most have not been rigorously tested in the health service systems of high‐income countries, limiting generalisability of findings.

Future research investigating potential solutions should involve consultation with a wide range of stakeholders, including families, ANC staff and practitioners, ANC services management, policy makers at multiple levels of government, and organizations and agencies with interest and expertise in particular barriers. Evaluations, such as those recently conducted with First Nations Australian women (McLachlan et al., 2022), will need to consider which strategies are socially acceptable for particular populations, which are most feasible, and effective. Further research could also be undertaken within a rapid cycle improvement strategy that would lead to changes that are both responsive and effective.

## 7 CONCLUSION

Although the traditional ANC system in Australia is intended to be universally accessible, women experiencing disadvantage encounter multiple and complex barriers to attendance. This study found ANC attendance barriers were often experienced concurrently and caused women considerable distress. Though many of the women in this study were able to muster sufficient resources to overcome these barriers at least some of the time, the findings indicate that the current system of ANC may not adequately support many disadvantaged women and their families. For these women, many barriers to accessing ANC stem from social determinants of health, where inequitable access will further exacerbate inequities. However, the findings also suggest several avenues to guide the development of specific strategies for increasing timely and adequate attendance at ANC consultations. The social determinants of health are complex and cannot be easily mitigated by single interventions such as ANC. Nonetheless, improving ANC attendance is an important contributory step toward more equitable health and development outcomes for Australian children, their families, and society more broadly.

## AUTHOR CONTRIBUTIONS

**Sarah V. Penman**: Conceptualization, methodology, investigation, data curation, formal analysis, writing—original draft, writing—review and editing. **Ruth M. Beatson**: Validation, Writing—review and editing. **Elizabeth H. Walker**: Formal analysis, writing—review and editing. **Sharon Goldfeld**: Funding acquisition, writing—review and editing. **Carly S. Molloy**: Funding acquisition, conceptualization, methodology, project administration, supervision, data curation, writing—review and editing. All authors have agreed on the final version and meet at least one of the following criteria (recommended by the ICMJE): (1) substantial contributions to conception and design, acquisition of data, or analysis and interpretation of data; (2) drafting the article or revising it critically for important intellectual content.

## FUNDING INFORMATION

This research was funded by the Paul Ramsay Foundation, and Eureka Benevolent Foundation. The research is also supported by the Victorian Government's Operational Infrastructure Support Program. Prof Goldfeld is supported by Australian National Health and Medical Research Council (NHMRC) Practitioner Fellowship (APP1155290). The funding bodies had no role in relation to the design and conduct of the study; collection, management, analysis, and interpretation of the data; preparation, review, or approval of the article; and decision to submit the article for publication.

## CONFLICT OF INTEREST STATEMENT

The authors have no conflicts of interest relevant to this article to disclose.

### PEER REVIEW

The peer review history for this article is available at https://www.webofscience.com/api/gateway/wos/peer‐review/10.1111/jan.15724.

## Data Availability

In compliance with ethics approval raw data is not publicly available. It will be stored electronically and then destroyed either 7 years following project completion or 5 years from publication of results.
